# Development and validation of a risk prediction model for perioperative acute kidney injury in non-cardiac and non-urological surgery patients: a retrospective cohort study

**DOI:** 10.3389/fphys.2025.1628450

**Published:** 2025-07-17

**Authors:** Xuhui Cong, Xuli Zou, Ruilou Zhu, Yubao Li, Lu Liu, Jiaqiang Zhang

**Affiliations:** ^1^Department of Anesthesia and Perioperative Medicine, Zhengzhou University People’s Hospital and Henan Provincial People’s Hospital, Zhengzhou, Henan, China; ^2^ Xinxiang Medical University, Xinxiang, Henan, China; ^3^ Zhengzhou University, Zhengzhou, Henan, China

**Keywords:** prediction model, risk assessment, perioperative acute kidney injury (AKI), non-cardiac, nonurological surgeries, clinical decision-making

## Abstract

**Background:**

This study presents a predictive model designed to fill the gap in tools for predicting perioperative acute kidney injury (AKI) in patients undergoing non-cardiac, non-urological surgeries, with the goal of improving clinical decision-making and patient outcomes.

**Methods:**

A retrospective cohort of 40,520 patients aged 65 and older who underwent non-cardiac, non-urological surgeries was analyzed. Key risk factors were identified using univariable logistic regression and LASSO, while multivariate logistic regression was applied to develop and validate the model.

**Results:**

The prediction model, based on 18 key variables including demographic data, comorbidities, and intraoperative factors, demonstrated strong discriminatory power for predicting perioperative AKI (AUC = 0.803; 95% CI, 0.783–0.823). It also showed a good fit in the validation cohort (Hosmer–Lemeshow test, χ^2^ = 5.895, *P* = 0.750). Decision curve analysis further confirmed the model’s significant clinical utility.

**Conclusion:**

This model effectively predicts perioperative AKI, providing a valuable tool for personalized risk assessment and prevention strategies in non-cardiac, non-urological surgeries. Further validation in diverse populations is recommended to optimize its clinical application.

## 1 Introduction

Perioperative acute kidney injury (AKI) is a serious complication of surgery, leading to prolonged hospital stays, higher healthcare costs, and increased postoperative complications and mortality. Studies have shown that factors such as the type of surgery, anesthesia techniques, intraoperative hypotension, blood loss, and fluid management can significantly affect renal function (Gumbert et al., 2020; Zarbock et al., 2023). According to the Kidney Disease: Improving Global Outcomes (KDIGO) guidelines, AKI is primarily diagnosed based on changes in serum creatinine levels and urine output. Intraoperative hypotension, ischemia-reperfusion injury, and drug toxicity can disrupt renal blood flow and cause tubular damage, contributing to AKI (Sharma and Slawski, 2018; Uusalo et al., 2021).

The development of perioperative AKI is also influenced by patient-specific factors such as age, baseline renal function, and comorbidities (e.g., diabetes, hypertension), as well as surgery-specific factors like the type and duration of the procedure. Additionally, intraoperative fluid management, anesthetic choices, and hemodynamic monitoring are critical in preventing and managing AKI (Sharma and Slawski, 2018; Uusalo et al., 2021). Predictive models integrating preoperative, intraoperative, and postoperative data have been developed to identify high-risk patients and guide personalized management strategies. While numerous studies have explored the mechanisms and risk factors of perioperative AKI, findings vary across different surgical types and patient populations. Further research into the pathophysiology of AKI, along with improvements in predictive models and their clinical application, is crucial for reducing AKI incidence and enhancing patient outcomes (Gumbert et al., 2020; Saugel et al., 2023).

## 2 Methods

### 2.1 Data sources and preprocessing

Data from the electronic medical records of patients treated at Henan Provincial People’s Hospital from June 2016 to June 2021 were retrospectively analyzed. Informed consent was waived by the Hospital’s Ethics Review Committee (Approval No. 2021-157), which determined that the study met the criteria for a waiver according to relevant guidelines. All methods adhered to these guidelines and regulations. The study was approved by the Ethics Review Committee (Approval No. 2021-157). The data focused on patients aged 65 and older who underwent non-cardiac, non-urological surgeries, regardless of perioperative acute kidney injury status. The dataset included patient demographics, comorbidities, and relevant laboratory findings for these surgical cases.

### 2.2 Study population

**FIGURE 1:**
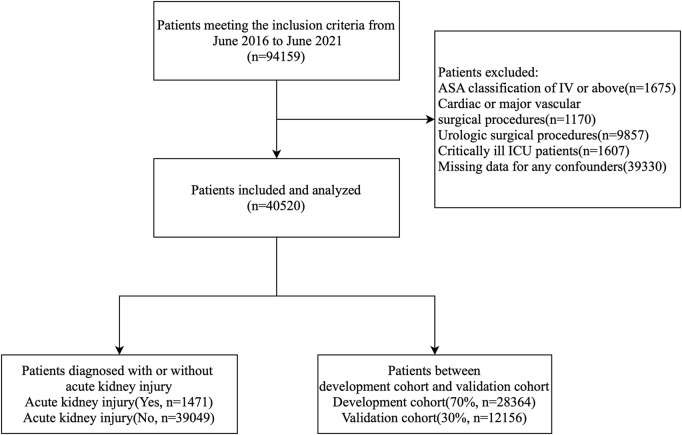
Patient flow diagram. ASA, American Society of Anesthesiologists.

The inclusion criteria for this study were: 1) patients who underwent non-cardiac and non-urological surgeries; 2) patients aged ≥65 years; and 3) patients with an ASA classification of I-III. The exclusion criteria were: 1) patients who underwent cardiac, major vascular, or urological surgeries; 2) patients with stage 5 chronic kidney disease; 3) critically ill patients in the ICU; 4) ASA classification IV-V; and 5) missing clinical or laboratory data. A total of 40,520 patient records were analyzed, with the data randomly divided into a development cohort and a validation cohort at a 70:30 ratio. Figure 1 illustrates the processing steps in detail.

### 2.3 Clinical variables and definitions

This study gathered perioperative demographic, clinical, imaging, and laboratory data from all patients, covering the period from 7 days before to 7 days after surgery.

The study considered the following variables:

1. Demographic characteristics: age, gender, ASA classification.
2. Surgery-related variables: type of surgery, duration of surgery, duration of anesthesia, type of anesthesia.
3. Comorbidities and medical history: smoking history, alcohol history, ascites, hypertension, diabetes, coronary artery disease, angina, valvular heart disease, myocardial infarction, heart failure, arrhythmia, atrial fibrillation, coronary stent implantation, cardiac surgery, peripheral vascular disease, COPD, dialysis, renal insufficiency, history of cerebrovascular disease, TIA, stroke, paraplegia, cancer.
4. Preoperative laboratory tests: white blood cell count, red blood cell count, platelet count, hemoglobin level, serum creatinine, serum albumin level, alanine aminotransferase, aspartate aminotransferase, blood sodium, blood potassium, blood calcium, thrombin time (TT), prothrombin time (PT), activated partial thromboplastin time (APTT), plasma prothrombin time, INR.
5. Preoperative medications: antihypertensives, ACE inhibitors, ARBs, calcium channel blockers, metoprolol, steroids, statins, diuretics, anticoagulants, antiplatelets, beta-blockers, nonsteroidal anti-inflammatory drugs, hypoglycemic drugs, insulin, aspirin and cessation days, clopidogrel and cessation days, heparin and cessation days, butylphthalide and duration of use, edaravone and duration of use, dextran and duration of use.
6. Intraoperative vital signs monitoring: blood pressure, heart rate, body temperature, BIS value, end-tidal carbon dioxide.
7. Intraoperative medications: inhaled anesthetics, propofol, sufentanil, remifentanil, nonsteroidal drugs, diuretics, anticoagulants, steroids, dexamethasone, methylprednisolone, dexmedetomidine.
8. Intraoperative fluid management, transfusions, and output: colloids, crystalloids, 0.9% sodium chloride, hydroxyethyl starch, succinyl gelatin, mannitol, glucose, albumin, red blood cells, plasma, platelets, cryoprecipitate, autologous blood, urine output, blood loss.
9. Intraoperative vasoactive drugs: ephedrine, adrenaline, norepinephrine, phenylephrine, dopamine, nitroglycerin, urapidil, nicardipine, clevidipine, phentolamine.
10. Postoperative medications: statins, anticoagulants, antiplatelets, dextran (dose and duration of use).

### 2.4 AKI diagnostic criteria

AKI was defined according to the Kidney Disease: Improving Global Outcomes (KDIGO) guidelines, which specify an increase in serum creatinine of ≥26.5 μmol/L within 48 h or a rise to ≥1.5 times the baseline level within 7 days postoperatively (de Boer et al., 2022).

### 2.5 Statistical analysis

We used various statistical methods to analyze both continuous and categorical variables. Continuous variables are expressed as medians with interquartile ranges (25th to 75th percentiles), with differences between groups assessed using the Mann–Whitney U test. Categorical variables are presented as frequencies and percentages, with differences evaluated using the chi-square test or Fisher’s exact test.

Logistic regression analysis was performed using the glmnet package in R, applying the LASSO method for variable selection. Significant variables were incorporated into the logistic regression model through forward stepwise selection to minimize the Akaike Information Criterion (AIC), ensuring inclusion of the fewest relevant variables.

#### 2.5.1 Assessment of multicollinearity

To assess multicollinearity, we performed multivariate logistic regression and calculated the variance inflation factor (VIF). A VIF value below 10 indicated no significant multicollinearity. We also created a correlation matrix heatmap to visualize relationships between variables and evaluated both VIF and tolerance values to assess collinearity.

#### 2.5.2 Handling missing data

We used Multiple Imputation by Chained Equations (MICE) to address missing data. MICE generates multiple imputed datasets by iteratively estimating missing values based on observed relationships among variables. This method provides a more accurate representation of missing data compared to simpler approaches like mean or median imputation. A sensitivity analysis compared the performance of models using the MICE-imputed dataset to models excluding missing data. Results confirmed that MICE minimally impacted model performance.

#### 2.5.3 Model performance evaluation

Model performance was evaluated using a diagnostic chart with significant variables. The receiver operating characteristic (ROC) curve assessed discrimination, with the area under the curve (AUC) as a measure of performance. Calibration was evaluated with calibration plots (Bootstrap method) and Spiegelhalter’s Z test, with good calibration indicated by close alignment between predicted and observed outcomes. Decision curve analysis (DCA) was performed, and net benefits were calculated at clinically relevant thresholds (e.g., 5%, 10%, 20%). Calibration was further assessed by grouping predicted probabilities into deciles and comparing the observed *versus* predicted AKI rates.

#### 2.5.4 Clinical application

To facilitate clinical use, a nomogram was developed as a visual tool for clinicians to predict patient outcomes based on the model.

#### 2.5.5 Development and validation cohorts

A random 70% sample of the cohort served as the development cohort, while the remaining 30% was used as the validation cohort. The rms package in R was used for graphical evaluation of model performance. All statistical tests were two-sided, with a significance threshold set at a p-value of less than 0.05. All analyses were performed using R version 4.3.1.

#### 2.5.6 Sensitivity analysis

To assess the robustness of model performance with respect to missing data, we performed a sensitivity analysis comparing models developed using Multiple Imputation by Chained Equations (MICE) and those based on complete-case analysis. Model evaluation was conducted using AUC, accuracy, sensitivity, specificity and F1 score.

To address concerns about certain variables reflecting perioperative severity or interventions, we conducted a sensitivity analysis. A secondary model using only preoperative variables (excluding intraoperative or postoperative factors) was developed and evaluated. This “preoperative-only” model was built using LASSO followed by multivariate logistic regression and compared to the primary model. Both models were assessed using AUC, accuracy, sensitivity, specificity, and F1 score.

Due to the retrospective nature of the dataset and the lack of standardized perioperative urine-output documentation, we were unable to conduct a sensitivity analysis based on the full KDIGO AKI definition. This represents a limitation of the current study. Future prospective studies should aim to collect complete urine output records to enable full diagnostic alignment with KDIGO criteria.

All code, synthetic data, and documentation required to reproduce the analyses are hosted in our GitHub repository (https://github.com/Iory-lab/Model-3.0). The exact version of the analytical code is archived under commit 842928e, while the corresponding documentation (README) is available under commit d7eb6be.

## 3 Results

In this study, we analyzed 1,471 perioperative AKI patients, divided into a development cohort of 1,029 patients and a validation cohort of 442 patients. A total of 151 clinical features were extracted, of which 42 contained missing data. The proportion of missing data for each feature was below 10% and 88.6% of the patients had complete data across all features. Descriptive statistical analyses are presented in Supplementary Tables 1, 2, with Supplementary Table 1 comparing patients with and without perioperative acute kidney injury, and Supplementary Table 2 contrasting the characteristics of the development and validation cohorts.

**FIGURE 2:**
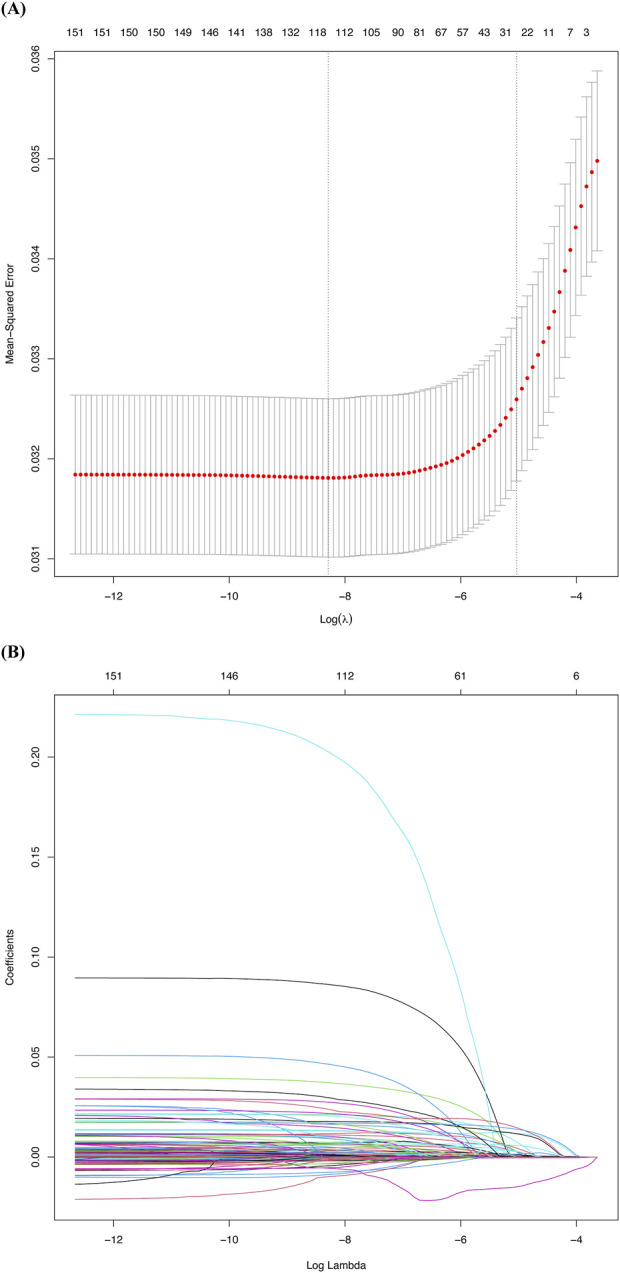
Factor selection using the least absolute shrinkage and selection operator (LASSO) logistic regression. **(A)** The LASSO coefficient profiles of the 151 candidate variables. A plot of the coefficient profile was generated against the log(λ). **(B)** Selection of the tuning parameter (λ) was performed using LASSO penalized logistic regression with 10-fold cross-validation.

**FIGURE 3:**
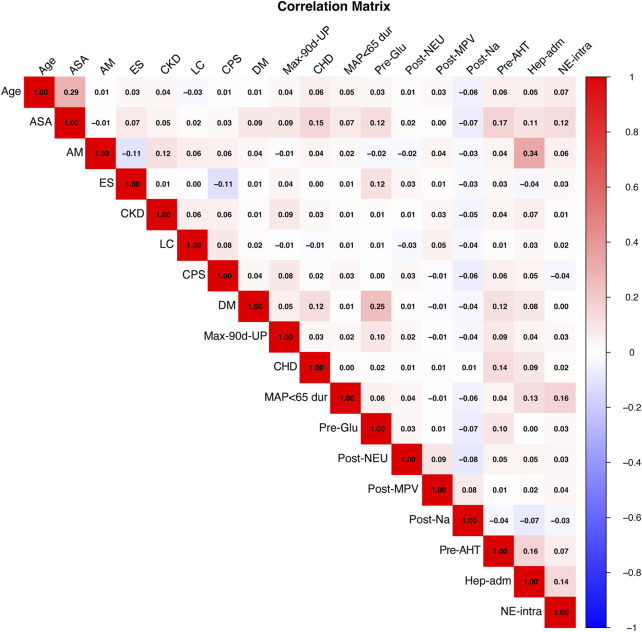
Heatmap of Pairwise Correlations Among Variables. This heatmap visualizes the pairwise correlations between the variables included in the model. Positive correlations are represented in red, while negative correlations are depicted in blue. The intensity of the color reflects the strength of the correlation, with darker shades indicating stronger correlations. ASA, American Society of Anesthesiologists; AM, Anesthesia methods; ES, Emergency surgery; CKD, Chronic Kidney Disease; LC, Liver cirrhosis; CPS, Child-Pugh score; DM, Diabetes; Max-90d-UP, Max 90-day pre-op urine protein; CHD, Coronary heart disease; MAP<65 dur, Mean arterial pressure <65 mmHg duration; Pre-Glu, Preoperative glucose; Post-NEU, Postoperative neutrophils; Post-MPV, Postoperative mean platelet volume measurement; Post-Na, Postoperative serum sodium; Pre-AHT, Preoperative antihypertensive medication; Hep-adm, Heparin during admission; NE-intra, Intraoperative norepinephrine.

To construct the predictive model, we used the LASSO logistic regression algorithm to select relevant features. Among the 151 features, 30 were selected based on nonzero coefficients, with an optimal lambda value of 0.0002754 (Figure 2). Multivariate logistic regression was then performed using these 30 factors. The best-fitting model was determined by comparing Akaike Information Criterion (AIC) values. Figure 3 and Table 1 display the correlation matrix, variance inflation factors (VIF), and tolerance values for each variable, respectively.

**TABLE 1:** Variance inflation factors (VIF) and corresponding tolerance values for each variable.

Variables	VIF	Tolerance
Age	1.101763	0.9076361
ASA	1.179344	0.8479291
Anesthesia methods	1.162552	0.8601763
Emergency surgery	1.045272	0.9566885
Chronic Kidney Disease	1.031904	0.9690825
Liver cirrhosis	1.021543	0.9789114
Child-Pugh score	1.042135	0.9595686
Diabetes	1.097386	0.911256
Max 90-day pre-op urine protein	1.036597	0.9646952
Coronary heart disease	1.055851	0.9471037
Mean arterial pressure <65 mmHg duration	1.048448	0.9537903
Preoperative glucose	1.110083	0.9008335
Postoperative neutrophils	1.026089	0.9745745
Postoperative mean platelet volume measurement	1.025862	0.9747896
Postoperative serum sodium	1.038448	0.9629753
Preoperative antihypertensive medication	1.084485	0.922097
Heparin during admission	1.208883	0.8272097
Intraoperative norepinephrine	1.061903	0.941706

Ultimately, the final model included 18 significant factors: age (OR: 1.04, 95% CI: 1.03–1.05), ASA classification (OR: 1.75, 95% CI: 1.51–2.01), anesthesia methods (OR: 1.59, 95% CI: 1.39–1.82), emergency surgery (OR: 1.63, 95% CI: 1.37–1.94), chronic kidney disease (CKD, OR: 1.69, 95% CI: 1.38–2.07), liver cirrhosis (OR: 1.63, 95% CI: 1.04–2.55), Child-Pugh score (OR: 1.28, 95% CI: 1.22–1.34), diabetes (OR: 1.27, 95% CI: 1.07–1.52), maximum 90-day preoperative urine protein (OR: 1.26, 95% CI: 1.14–1.4), coronary heart disease (OR: 1.42, 95% CI: 1.19–1.7), mean arterial pressure <65 mmHg duration (OR: 1.01, 95% CI: 1.01–1.01), preoperative glucose (OR: 1.07, 95% CI: 1.04–1.09), postoperative neutrophils (OR: 1.04, 95% CI: 1.03–1.06), postoperative mean platelet volume (OR: 1.25, 95% CI: 1.19–1.31), postoperative serum sodium (OR: 1.04, 95% CI: 1.03–1.06), preoperative antihypertensive medication (OR: 1.38, 95% CI: 1.2–1.58), heparin during admission (OR: 6.1, 95% CI: 4.34–8.58), and intraoperative norepinephrine (OR: 1.77, 95% CI: 1.51–2.08) (Table 2).

**TABLE 2:** Multivariate logistic regression analysis for risk factors associated with acute kidney injury in perioperative non-cardiac and non-urological surgery patients.

Variables	B (original)	B (adjusted)	SE	OR	CI	Z	*P*
Intercept	−20.134	−20.155	1.196	0	0–0	−16.835	0
Age	0.042	0.042	0.006	1.04	1.03–1.05	7.571	0
ASA	0.557	0.56	0.073	1.75	1.51–2.01	7.655	0
Anesthesia methods	0.461	0.463	0.069	1.59	1.39–1.82	6.647	0
Emergency surgery [Yes]	0.489	0.487	0.088	1.63	1.37–1.94	5.548	0
Chronic Kidney Disease (CKD) [Yes]	0.523	0.516	0.104	1.69	1.38–2.07	5.02	0
Liver cirrhosis [Yes]	0.487	0.465	0.229	1.63	1.04–2.55	2.13	0.033
Child-Pugh score	0.249	0.250	0.024	1.28	1.22–1.34	10.447	0
Diabetes [Yes]	0.241	0.244	0.089	1.27	1.07–1.52	2.707	0.007
Max 90-day pre-op urine protein	0.232	0.234	0.053	1.26	1.14–1.4	4.354	0
Coronary heart disease [Yes]	0.35	0.348	0.091	1.42	1.19–1.7	3.856	0
Mean arterial pressure <65 mmHg duration	0.008	0.008	0.001	1.01	1.01–1.01	6.856	0
Preoperative glucose	0.064	0.063	0.013	1.07	1.04–1.09	4.745	0
Postoperative neutrophils	0.044	0.044	0.007	1.04	1.03–1.06	5.993	0
Postoperative mean platelet volume measurement	0.222	0.222	0.025	1.25	1.19–1.31	8.742	0
Postoperative serum sodium	0.042	0.042	0.008	1.04	1.03–1.06	5.402	0
Preoperative antihypertensive medication [Yes]	0.32	0.32	0.07	1.38	1.2–1.58	4.578	0
Heparin during admission [Yes]	1.808	1.82	0.174	6.1	4.34–8.58	10.372	0
Intraoperative norepinephrine [Yes]	0.572	0.574	0.083	1.77	1.51–2.08	6.905	0

Abbreviations: ASA, american society of anesthesiologists; B, regression coefficient; SE, standard error; OR, odds radio; CI, credibility interval.

**FIGURE 4:**
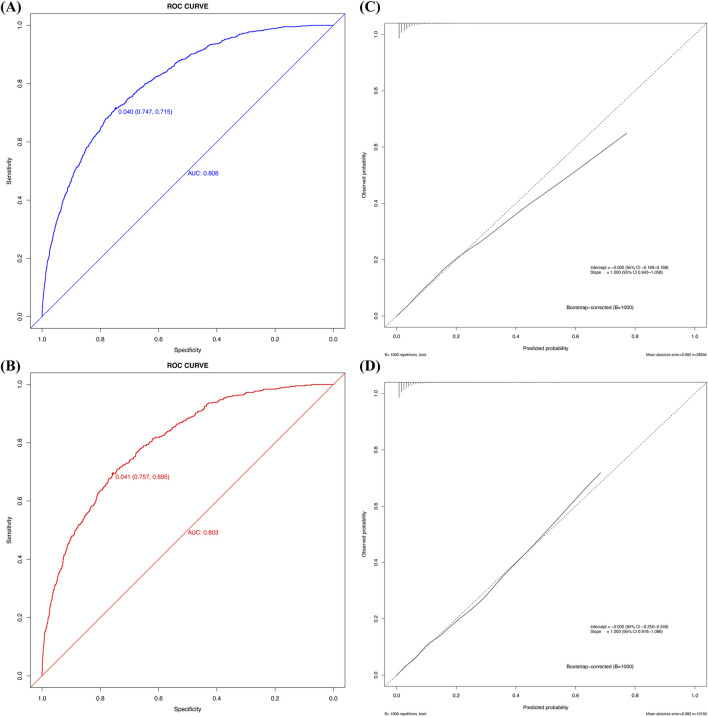
The ROC curve, calibration plot of the new predictive model. **(A)** ROC curve of the new predictive model with the development cohort; **(B)** ROC curve for the new prediction model with the validation cohort; **(C)** Calibration plot of the new predictive model with the development cohort; **(D)** Calibration plot of the new predictive model with the validation cohort.

Cross-validation was performed in both cohorts using R Studio, yielding AUC values for the model. In the development cohort, the AUC was 0.808 (95% CI 0.795–0.820, *P* < 0.05) with a C-index of 0.808, while the validation cohort achieved an AUC of 0.803 (95% CI 0.783–0.823, *P* < 0.05) with a C-index of 0.803 (Figure 4). Sensitivity analysis of the missing data treatment method showed no significant impact on model performance, indicating that the imputation approach did not significantly affect the results (Table 3).

**TABLE 3:** Comparative performance of different models based on AUC, accuracy, sensitivity, specificity, and F1 score.

Model	AUC	Accuracy	Sensitivity	Specificity	F1 score
Model with Imputed Data	0.813 (95% CI: 0.793–0.8333)	0.966	0.9991	0.03	0.983
Model with Complete Case Analysis	0.808 (95% CI: 0.788–0.828)	0.964	0.999	0.0381	0.982
Preoperative Variables Model	0.768 (95% CI: 0.747–0.789)	0.963	0.9997	0	0.981

**FIGURE 5:**
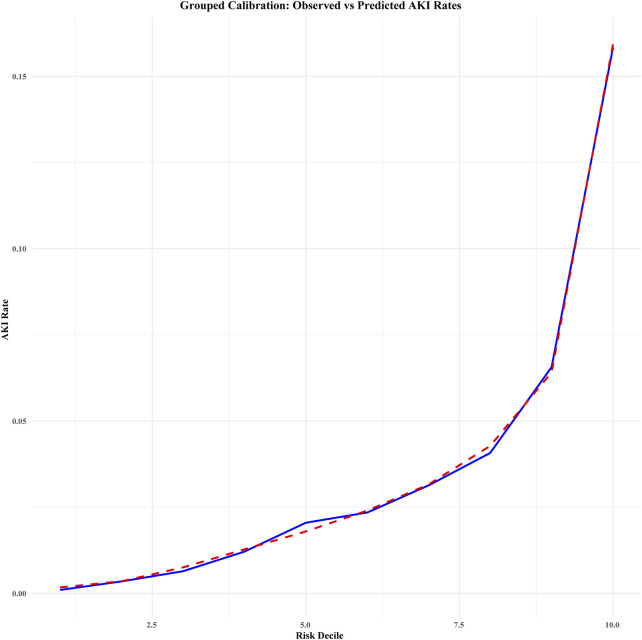
Grouped calibration of observed vs. predicted acute kidney injury (AKI) rates across risk deciles (combined data). This calibration plot compares the observed and predicted rates of acute kidney injury (AKI) across deciles of predicted risk. The blue solid line represents the observed AKI rates (i.e., the actual incidence) within each risk decile, while the red dashed line illustrates the predicted AKI rates (i.e., the model’s estimated probability) for each decile. Deciles were determined based on the predicted risk scores from the model, and the plot provides an assessment of the model’s calibration in predicting AKI. The legend distinguishes between observed and predicted AKI rates for clarity.

Calibration curve analysis confirmed strong agreement between predicted and actual probabilities in both cohorts, with Brier scores of 0.032 for both the development and validation cohorts. Furthermore, the Hosmer–Lemeshow test indicated good model fit, with P-values greater than 0.05 in both cohorts (development cohort χ^2^ = 4.215, *P* = 0.900; validation cohort χ^2^ = 5.895, *P* = 0.750). Figure 4 illustrates the calibration curve after bootstrap optimism correction with 1000 iterations, demonstrating a strong alignment between predicted and observed probabilities. The corrected intercept and slope, clearly marked on the curve, provide additional evidence of the model’s robust calibration. The adjusted AUC of 0.8047 further corroborates the model’s stability and predictive accuracy across both the development and validation cohorts. Grouped calibration analysis demonstrated close agreement between observed and predicted event rates across all risk deciles (Figure 5), further supporting the model’s calibration. Together, these results highlight the model’s exceptional performance and its potential utility for clinical decision-making in the prediction of perioperative acute kidney injury (AKI).

A sensitivity analysis was conducted to assess the effect of perioperative variables, such as heparin during admission and intraoperative norepinephrine, by excluding intraoperative and postoperative data. The results, presented in Table 3, showed that the full model (including perioperative variables) achieved an AUC of 0.813, with high accuracy (96.6%) and sensitivity (99.91%). In contrast, the preoperative-only model showed a lower AUC of 0.768, with sensitivity of 99.97%, but lacked specificity (0%). These results indicate that the full model offers the best balance of sensitivity, specificity, and clinical relevance, supporting its selection as the preferred model for perioperative AKI risk prediction.

**FIGURE 6:**
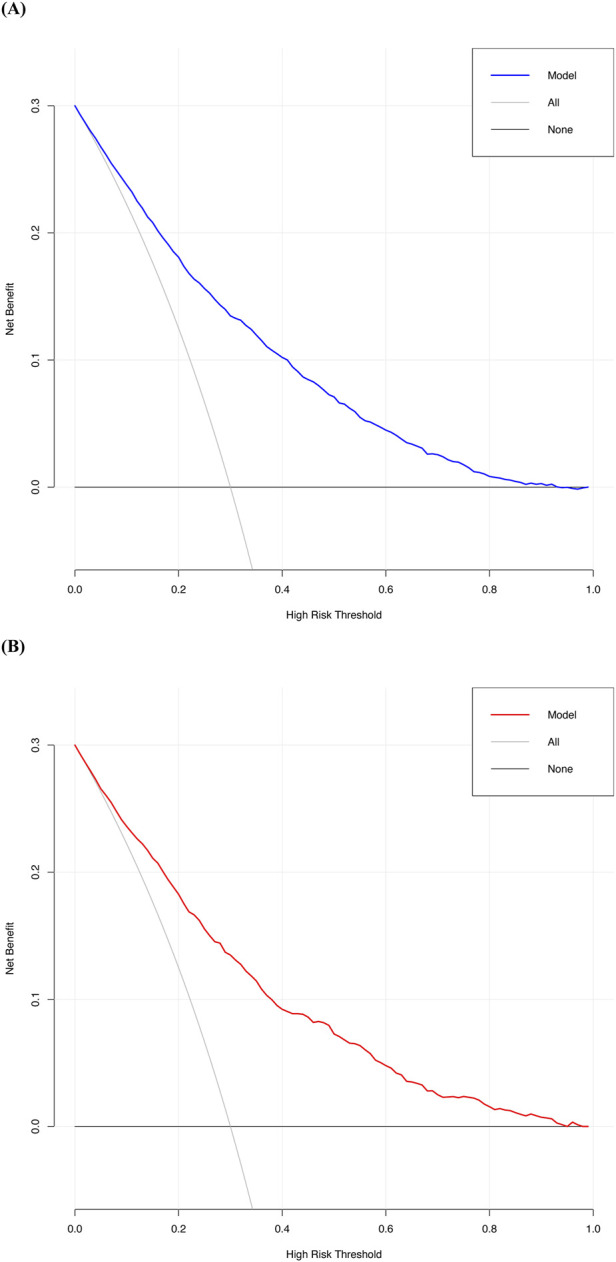
The decision curve of the new predictive model. **(A)** Decision curve of the new predictive model with the development cohort; **(B)** Decision curve of the new predictive model with the validation cohort.

Decision curve analysis revealed that the threshold probabilities for clinical intervention to prevent perioperative AKI in patients undergoing non-cardiac, non-urological surgeries ranged from 0% to 90% in the development cohort and 0%–94% in the validation cohort. These findings suggest that clinical interventions could be effective within these probability ranges (Figure 6). Although the predicted risk of AKI in the general perioperative population is typically less than 15%, the inclusion of decision thresholds up to 90% allows us to evaluate the model’s performance in high-risk scenarios, particularly for patients with the highest predicted risk, ensuring that these individuals are prioritized for timely interventions. Additionally, net benefit values at clinically relevant thresholds (e.g., 5%, 10%, and 20%) are presented in Table 4. These thresholds better represent realistic perioperative AKI rates, offering a more accurate understanding of the model’s practical utility. The net benefit analysis at these thresholds helps to strike an appropriate balance between true positives and false positives, demonstrating the model’s applicability in clinical decision-making for patients at varying levels of risk.

**TABLE 4:** Net benefit of the prediction model at clinically relevant risk thresholds.

Risk threshold	Net benefit (95% CI)
5% (0.05)	0.892 (0.885, 0.898)
10% (0.10)	0.792 (0.780, 0.802)
20% (0.20)	0.603 (0.579, 0.622)

Net benefit values were derived from decision curve analysis, representing the clinical benefit of the model compared to universal treatment or no treatment. A threshold probability of 5%, 10%, and 20% was selected based on typical clinical decision points for perioperative acute kidney injury (AKI) risk stratification.

**FIGURE 7:**
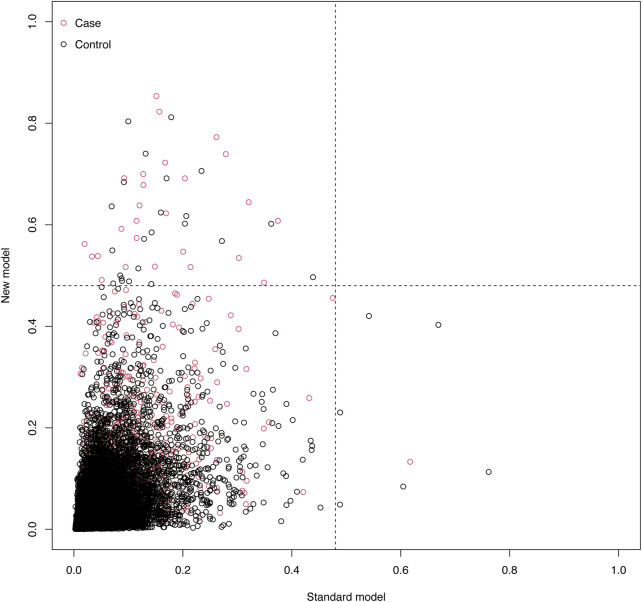
Scatter Plot of Net Reclassification Improvement (NRI) Between Models. This plot compares the NRI of the standard model (x-axis) and the new model (y-axis). Red dots represent cases, and black dots represent controls. The event probability ranges from 0 to 1, illustrating the risk reclassification between the two models.

To benchmark the new model, we compared its performance with the SPARK score, an established perioperative AKI risk prediction tool. In the validation cohort, the SPARK score achieved an AUC of 0.763 (95% CI: 0.741–0.763), a Brier score of 0.033, and a Hosmer–Lemeshow test χ^2^ of 8.63 (*P* = 0.472), indicating a satisfactory model fit. In contrast, the new model outperformed the SPARK score, achieving an AUC of 0.803 (95% CI: 0.783–0.823), reflecting better discriminative ability. Additionally, Net Reclassification Improvement (NRI) analysis showed that the new model enhanced risk classification, with an overall NRI of 0.0237 (95% CI: 0.01137–0.04087), indicating a 2.37% improvement in accurate reclassification. The new model was particularly effective in identifying high-risk patients (NRI+ = 0.0243, 95% CI: 0.01184–0.04171), while minimizing the misclassification of healthy patients as high-risk (NRI- = −0.0006, 95% CI: −0.00128 to −0.00022). These findings underscore the new model’s superior accuracy in identifying high-risk patients and reducing false positives, thus enhancing its clinical utility (Figure 7).

**FIGURE 8:**
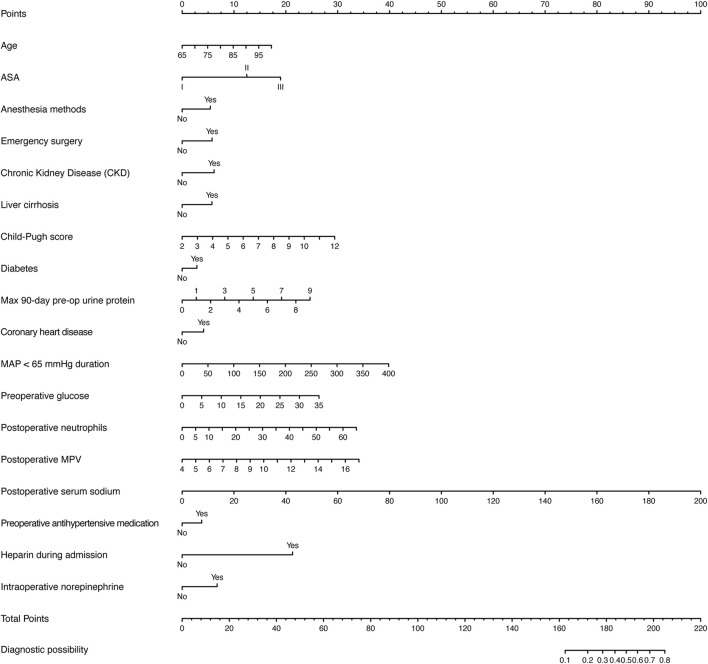
Nomogram of the logistic regression model for perioperative AKI risk prediction.

Figure 8 presents a nomogram illustrating the logistic model. Age, duration of MAP <65 mmHg, preoperative glucose, postoperative neutrophils, postoperative MPV, and postoperative serum sodium are treated as continuous variables, while the remaining variables are categorical. Based on a Youden index of 0.40, the optimal cutoff value on the nomogram was determined to be 185, yielding high sensitivity (71.5%) and specificity (74.7%). Scores above 185 indicate a high risk of perioperative AKI.

After validating the model’s discrimination, calibration, and clinical decision-making abilities, we confirmed its clinical effectiveness. The analysis of patient characteristics and AKI risk factors facilitated the development of a multivariate logistic regression model, which was rigorously validated and assessed using various graphical tools.

## 4 Discussion

Perioperative acute kidney injury (AKI) is a common and serious complication in surgical patients, particularly in high-risk procedures like cardiac and organ transplantation surgeries, where the incidence can reach 30%–50% (Zarbock et al., 2015). AKI not only prolongs hospital stays and increases healthcare costs but also significantly raises postoperative mortality and the risk of chronic kidney disease (CKD) and end-stage renal disease (ESRD) (Hoste et al., 2015). Despite advances in perioperative management, the incidence of AKI remains high, highlighting the challenges of accurate prediction and timely intervention during this critical period (Khwaja, 2012). Traditional risk scoring systems, such as RIFLE, AKIN, and KDIGO, primarily rely on postoperative renal function changes and have limited predictive ability before and during surgery, restricting their clinical utility (Forni et al., 2017). However, with the advent of machine learning and advanced statistical techniques, multifactorial prediction models incorporating a wide range of clinical variables have significantly enhanced AKI risk prediction accuracy, thereby supporting clinical decision-making and personalized prevention strategies (Coca et al., 2012).

This study developed a perioperative AKI prediction model specifically for non-cardiac and non-urological surgery patients, aiming to better isolate and analyze common risk factors within a more homogeneous cohort. Cardiac surgeries, such as those involving cardiopulmonary bypass, and urological surgeries, which directly affect renal perfusion, introduce unique variables that complicate risk factor identification. By excluding these two types of surgeries, we sought to create a model with broader applicability across various surgical settings. The results showed that the full model, incorporating perioperative variables, achieved an AUC of 0.813, with high accuracy (96.6%) and sensitivity (99.91%) (Ellis et al., 2018). I In comparison, the preoperative-only model had a lower AUC of 0.768, high sensitivity (99.97%), but lacked specificity (0%) (Guan et al., 2022). These findings suggest that while preoperative variables can identify high-risk patients, the full model offers the best balance of sensitivity, specificity, and clinical relevance, making it the preferred choice for perioperative AKI prediction (Chen et al., 2024).

To improve prediction further, we developed the model using LASSO and multivariate logistic regression, which offered significant advantages. The LASSO algorithm selected 30 key variables, reducing the risk of overfitting and enhancing model robustness (Tomašev et al., 2019). Logistic regression further narrowed these variables down to 18, encompassing patient characteristics, surgical type, and biomarkers, which provided a comprehensive risk assessment (Tomašev et al., 2019). The model achieved AUC values of 0.808 and 0.803 in the development and validation cohorts, respectively, indicating good discriminative ability. The Brier score of 0.032 confirmed the consistency between predicted and actual outcomes (Khwaja, 2012).

Decision curve analysis demonstrated that the model offered substantial clinical benefit across a wide range of threshold probabilities (0%–90% in the development cohort and 0%–94% in the validation cohort). Notably, as shown in Table 4, the model provided significant net benefit at clinically relevant risk thresholds (5%, 10%, and 20%), effectively identifying high-risk patients (Zarbock et al., 2015). These features make the model a valuable tool for perioperative management, assisting clinicians in implementing precise, individualized interventions.

The perioperative AKI prediction model developed in this study incorporates 18 key factors spanning the preoperative, intraoperative, and postoperative stages, emphasizing the multifactorial nature of AKI. Preoperatively, elderly patients are at higher risk of AKI due to declining renal function, high ASA scores, cardiovascular disease, diabetes, and chronic kidney disease (Anderson et al., 2011). Elevated preoperative urine protein, hyperglycemia, and coronary heart disease further heighten the risk by impairing renal perfusion (Coca et al., 2012). Additionally, antihypertensive medications, which can affect renal blood flow, increase the likelihood of AKI (Löffler and Bourque, 2016).

Intraoperatively, fluctuations in blood pressure and reduced cardiac output—especially in patients undergoing general anesthesia or emergency surgery—substantially increase the risk of AKI, particularly in patients with liver cirrhosis (Sun et al., 2015). The risk is greater with higher Child-Pugh scores (Marik and Bellomo, 2013). Although norepinephrine is used to maintain blood pressure during surgery, its vasoconstrictive effects may reduce renal blood flow, thus increasing the risk of renal ischemia (Singer et al., 2016). Furthermore, the duration of intraoperative hypotension, particularly the cumulative time spent with a mean arterial pressure below 65 mmHg, is a critical determinant of AKI risk (Sun et al., 2015). This variable was incorporated into our final model to reflect the significant impact of both the severity and duration of intraoperative hypotension on renal perfusion and subsequent injury (Shaw et al., 2022).

Postoperatively, inflammatory responses and coagulation system activation (e.g., increased neutrophil count and mean platelet volume) further elevate the risk of AKI (Zarbock et al., 2015). Abnormal serum sodium levels, which indicate fluid imbalance, also contribute to this increased risk (Sterns, 2015). Together, these factors heighten the risk of perioperative AKI, underscoring the need for close monitoring and early intervention in clinical management (Kellum et al., 2021).

In comparison to existing perioperative AKI risk prediction models, such as the SPARK score, the new model showed superior performance in both the development and validation cohorts, with an AUC of 0.803 (vs. 0.763 for SPARK). NRI analysis further demonstrated that our model more accurately reclassified high-risk patients and reduced the misclassification of healthy patients, highlighting its clinical advantages. The higher AUC and favorable NRI results suggest a better balance of sensitivity and specificity, enabling more timely interventions and potentially improving patient outcomes. Given these advantages, the new model holds promise for integration into clinical practice as a reliable tool for preventing and managing perioperative AKI.

This study successfully developed an effective model for predicting perioperative AKI based on various clinical variables. The model performed well in both the development and validation cohorts, exhibiting high discrimination and calibration, and accurately identifying high-risk patients. These findings provide valuable guidance for preventing and managing perioperative AKI.

However, this study has several limitations. First, the model is based on single-center data, which may introduce sample selection bias and limit the generalizability of the results (Coca et al., 2012). Second, although the model includes several clinical variables related to AKI, it does not account for key factors such as intraoperative fluid management (Semler et al., 2018), blood pressure fluctuations (Salmasi et al., 2017), and intraoperative medication use. Third, the study was limited to patients aged 65 and older, a population at higher risk for perioperative AKI due to age-related decline in renal function (Shen et al., 2022). While this age group is critical for perioperative AKI prediction, excluding younger adults may limit the model’s applicability to mixed-age surgical populations (Gomelsky et al., 2020). Future studies should validate the model in multicenter cohorts and incorporate additional clinical variables to further optimize its performance.

Another limitation that requires attention is the potential for outcome misclassification. In this study, acute kidney injury (AKI) was defined solely by serum creatinine criteria, while urine output data—an essential component of the KDIGO definition—were unavailable. This reliance on serum creatinine alone for diagnosing AKI may introduce bias. Without urine output data, some patients who developed AKI but whose serum creatinine levels did not meet the predefined criteria may have been overlooked (Weiss et al., 2019). Conversely, patients with elevated serum creatinine levels but normal urine output—who may not have actual AKI—could have been misclassified (Fu et al., 2022). As a result, the reported incidence of perioperative AKI in patients undergoing non-cardiac and non-urological surgeries may be either underestimated or overestimated, deviating from the true prevalence (Schiefer et al., 2023).

To address this issue, future research should prioritize collecting complete urine-output data to minimize classification bias. Unfortunately, due to data limitations in the current study, conducting a sensitivity analysis restricted to patients with reliable urine-output records was not feasible. Additionally, we did not collect data on ischemic time, which is primarily relevant to vascular and cardiac surgeries that were excluded from this research. However, we did collect data on surgical and anesthesia durations, which may serve as indirect surrogates for ischemic time, particularly in surgeries where blood flow restriction may occur. This limitation has been addressed in the manuscript (Shaw et al., 2022; Zamirpour et al., 2023).

In the present study, AKI was defined solely based on serum creatinine criteria due to the unavailability of urine output data. According to KDIGO guidelines, this approach may lead to under- or overestimation of true AKI incidence (Kellum and Lameire, 2013). Prior studies have suggested that omitting urine output criteria can lead to a 20%–40% under-ascertainment of AKI events, especially in the early postoperative period. For example, in the PERISCOPE cohort, urine output alone accounted for nearly half of AKI diagnoses missed by creatinine alone (Göcze et al., 2018). Based on our observed AKI incidence of 3.6%, we estimate the actual rate could range between 4.3% and 5.0%, suggesting a relative underestimation of 28%–39% (Ostermann et al., 2020).

Furthermore, relying solely on serum creatinine may bias the effect estimates for certain intraoperative hemodynamic predictors. For instance, transient hypotension or vasopressor use—such as prolonged MAP <65 mmHg or intraoperative norepinephrine administration—may cause renal hypoperfusion leading to oliguria, but without immediate creatinine elevation. These subclinical cases may not be captured under a creatinine-only AKI definition, potentially attenuating the observed associations for these variables (Khwaja, 2012; Sun et al., 2015). As such, the impact of short-term hypotension on AKI risk may be underestimated, resulting in residual bias toward the null hypothesis.

To mitigate classification bias, future studies with larger sample sizes and data from multiple centers should aim to gather comprehensive urine-output data and conduct sensitivity analyses. This approach will help accurately assess the role of urine output in AKI diagnosis and improve the performance of prediction models, ultimately enhancing the validity and reliability of future research in this area (Neyra et al., 2023; Zamirpour et al., 2023).

Despite these limitations, the model demonstrated strong performance in predicting perioperative AKI. However, to extend its applicability, further validation in larger, more diverse populations is needed, along with refinement to include additional clinical factors.

## 5 Conclusion

This study identified key risk factors for perioperative acute kidney injury (AKI) in patients undergoing non-cardiac and non-urological surgeries, providing a foundation for improved management and prevention strategies. A predictive model was developed and validated, demonstrating strong performance and potential as a decision-support tool for clinicians. These findings lay the groundwork for future large-scale prospective trials to further validate the model and broaden its clinical application. Nonetheless, given the reliance on creatinine-only criteria, the model may be subject to misclassification bias, especially in cases of transient or oliguria-predominant AKI. Future work with comprehensive urine-output data is warranted to further enhance diagnostic accuracy.

## Data Availability

The raw data supporting the conclusions of this article will be made available by the authors, without undue reservation.

## References

[B1] Anderson S. Eldadah B. Halter J. B. Hazzard W. R. Himmelfarb J. Horne F. M. (2011). Acute kidney injury in older adults. J. Am. Soc. Nephrol. 22 (1), 28–38. 10.1681/asn.2010090934 21209252

[B2] Chen Y. Teng Y. Peng X. Zhu T. Liu J. Ou M. (2024). Combination of creatinine with inflammatory biomarkers (PCT, CRP, hsCRP) for predicting postoperative ICU admissions for elderly patients. Adv. Ther. 41 (7), 2776–2790. 10.1007/s12325-024-02874-1 38743240 PMC11213804

[B3] Coca S. G. Singanamala S. Parikh C. R. (2012). Chronic kidney disease after acute kidney injury: a systematic review and meta-analysis. Kidney Int. 81 (5), 442–448. 10.1038/ki.2011.379 22113526 PMC3788581

[B4] de Boer I. H. Khunti K. Sadusky T. Tuttle K. R. Neumiller J. J. Rhee C. M. (2022). Diabetes management in chronic kidney disease: a consensus report by the american diabetes association (ADA) and kidney disease: improving global outcomes (KDIGO). Diabetes Care 45 (12), 3075–3090. 10.2337/dci22-0027 36189689 PMC9870667

[B5] Ellis R. J. Del Vecchio S. J. Kalma B. Ng K. L. Morais C. Francis R. S. (2018). Association between preoperative hydration status and acute kidney injury in patients managed surgically for kidney tumours. Int. Urol. Nephrol. 50 (7), 1211–1217. 10.1007/s11255-018-1901-2 29869744

[B6] Forni L. G. Darmon M. Ostermann M. Oudemans-van Straaten H. M. Pettilä V. Prowle J. R. (2017). Renal recovery after acute kidney injury. Intensive Care Med. 43 (6), 855–866. 10.1007/s00134-017-4809-x 28466146 PMC5487594

[B7] Fu J. Kosaka J. Morimatsu H. (2022). Impact of different KDIGO criteria on clinical outcomes for early identification of acute kidney injury after non-cardiac surgery. J. Clin. Med. 11 (19), 5589. 10.3390/jcm11195589 36233456 PMC9571209

[B8] Göcze I. Jauch D. Götz M. Kennedy P. Jung B. Zeman F. (2018). Biomarker-guided intervention to prevent acute kidney injury after major surgery: the prospective randomized BigpAK study. Ann. Surg. 267 (6), 1013–1020. 10.1097/sla.0000000000002485 28857811

[B9] Gomelsky A. Abreo K. Khater N. Abreo A. Amin B. Craig M. K. (2020). Perioperative acute kidney injury: stratification and risk reduction strategies. Best. Pract. Res. Clin. Anaesthesiol. 34 (2), 167–182. 10.1016/j.bpa.2020.04.003 32711827

[B10] Guan H. L. Liu H. Hu X. Y. Abdul M. Dai M. S. Gao X. (2022). Urinary albumin creatinine ratio associated with postoperative delirium in elderly patients undergoing elective non-cardiac surgery: a prospective observational study. CNS Neurosci. Ther. 28 (4), 521–530. 10.1111/cns.13717 34415671 PMC8928921

[B11] Gumbert S. D. Kork F. Jackson M. L. Vanga N. Ghebremichael S. J. Wang C. Y. (2020). Perioperative acute kidney injury. Anesthesiology 132 (1), 180–204. 10.1097/aln.0000000000002968 31687986 PMC10924686

[B12] Hoste E. A. Bagshaw S. M. Bellomo R. Cely C. M. Colman R. Cruz D. N. (2015). Epidemiology of acute kidney injury in critically ill patients: the multinational AKI-EPI study. Intensive Care Med. 41 (8), 1411–1423. 10.1007/s00134-015-3934-7 26162677

[B13] Kellum J. A. Lameire N. KDIGO AKI Guideline Work Group (2013). Diagnosis, evaluation, and management of acute kidney injury: a KDIGO summary (part 1). Crit. Care 17 (1), 204. 10.1186/cc11454 23394211 PMC4057151

[B14] Kellum J. A. Romagnani P. Ashuntantang G. Ronco C. Zarbock A. Anders H. J. (2021). Acute kidney injury. Nat. Rev. Dis. Prim. 7 (1), 52. 10.1038/s41572-021-00284-z 34267223

[B15] Khwaja A. (2012). KDIGO clinical practice guidelines for acute kidney injury. Nephron Clin. Pract. 120 (4), c179–c184. 10.1159/000339789 22890468

[B16] Löffler A. I. Bourque J. M. (2016). Coronary microvascular dysfunction, microvascular angina, and management. Curr. Cardiol. Rep. 18 (1), 1. 10.1007/s11886-015-0682-9 26694723 PMC4835180

[B17] Marik P. E. Bellomo R. (2013). Stress hyperglycemia: an essential survival response. Crit. Care 17 (2), 305. 10.1186/cc12514 23470218 PMC3672537

[B18] Neyra J. A. Ortiz-Soriano V. Liu L. J. Smith T. D. Li X. Xie D. (2023). Prediction of mortality and major adverse kidney events in critically ill patients with acute kidney injury. Am. J. Kidney Dis. 81 (1), 36–47. 10.1053/j.ajkd.2022.06.004 35868537 PMC9780161

[B19] Ostermann M. Zarbock A. Goldstein S. Kashani K. Macedo E. Murugan R. (2020). Recommendations on acute kidney injury biomarkers from the acute disease quality initiative consensus conference: a consensus statement. JAMA Netw. Open 3 (10), e2019209. 10.1001/jamanetworkopen.2020.19209 33021646

[B20] Salmasi V. Maheshwari K. Yang D. Mascha E. J. Singh A. Sessler D. I. (2017). Relationship between intraoperative hypotension, defined by either reduction from baseline or absolute thresholds, and acute kidney and myocardial injury after noncardiac surgery: a retrospective cohort analysis. Anesthesiology 126 (1), 47–65. 10.1097/aln.0000000000001432 27792044

[B21] Saugel B. Hoste E. Chew M. S. (2023). A global perspective on acute kidney injury after major surgery: much needed insights and sobering results. Intensive Care Med. 49 (12), 1508–1510. 10.1007/s00134-023-07250-1 37906259 PMC10709254

[B22] Schiefer J. Bernardi M. H. Lichtenegger P. Schak G. Atallah L. Ristl R. (2023). Incidence and outcomes of AKI in postoperative patients admitted to ICU using full KDIGO criteria - a cohort study. J. Clin. Anesth. 89, 111156. 10.1016/j.jclinane.2023.111156 37356195

[B23] Semler M. W. Self W. H. Wanderer J. P. Ehrenfeld J. M. Wang L. Byrne D. W. (2018). Balanced crystalloids *versus* saline in critically ill adults. N. Engl. J. Med. 378 (9), 829–839. 10.1056/NEJMoa1711584 29485925 PMC5846085

[B24] Sharma K. Slawski B. (2018). Renal disease and the surgical patient: minimizing the impact. Cleve Clin. J. Med. 85 (7), 559–567. 10.3949/ccjm.85a.17009 30004381

[B25] Shaw A. D. Khanna A. K. Smischney N. J. Shenoy A. V. Boero I. J. Bershad M. (2022). Intraoperative hypotension is associated with persistent acute kidney disease after noncardiac surgery: a multicentre cohort study. Br. J. Anaesth. 129 (1), 13–21. 10.1016/j.bja.2022.03.027 35595549

[B26] Shen J. Chu Y. Wang C. Yan S. (2022). Risk factors for acute kidney injury after major abdominal surgery in the elderly aged 75 years and above. BMC Nephrol. 23 (1), 224. 10.1186/s12882-022-02822-7 35739472 PMC9229523

[B27] Singer M. Deutschman C. S. Seymour C. W. Shankar-Hari M. Annane D. Bauer M. (2016). The third international consensus definitions for sepsis and septic shock (Sepsis-3). Jama 315 (8), 801–810. 10.1001/jama.2016.0287 26903338 PMC4968574

[B28] Sterns R. H. (2015). Disorders of plasma sodium--causes, consequences, and correction. N. Engl. J. Med. 372 (1), 55–65. 10.1056/NEJMra1404489 25551526

[B29] Sun L. Y. Wijeysundera D. N. Tait G. A. Beattie W. S. (2015). Association of intraoperative hypotension with acute kidney injury after elective noncardiac surgery. Anesthesiology 123 (3), 515–523. 10.1097/aln.0000000000000765 26181335

[B30] Tomašev N. Glorot X. Rae J. W. Zielinski M. Askham H. Saraiva A. (2019). A clinically applicable approach to continuous prediction of future acute kidney injury. Nature 572 (7767), 116–119. 10.1038/s41586-019-1390-1 31367026 PMC6722431

[B31] Uusalo P. Hellman T. Järvisalo M. J. (2021). Mortality and associated risk factors in perioperative acute kidney injury treated with continuous renal replacement therapy. Perioper. Med. (Lond) 10 (1), 57. 10.1186/s13741-021-00227-y 34903294 PMC8670067

[B32] Weiss R. Meersch M. Pavenstädt H. J. Zarbock A. (2019). Acute kidney injury: a frequently underestimated problem in perioperative medicine. Dtsch. Arztebl Int. 116 (49), 833–842. 10.3238/arztebl.2019.0833 31888797 PMC6962766

[B33] Zamirpour S. Hubbard A. E. Feng J. Butte A. J. Pirracchio R. Bishara A. (2023). Development of a machine learning model of postoperative acute kidney injury using non-invasive time-sensitive intraoperative predictors. Bioeng. (Basel) 10 (8), 932. 10.3390/bioengineering10080932 PMC10451203 37627817

[B34] Zarbock A. Schmidt C. Van Aken H. Wempe C. Martens S. Zahn P. K. (2015). Effect of remote ischemic preconditioning on kidney injury among high-risk patients undergoing cardiac surgery: a randomized clinical trial. Jama 313 (21), 2133–2141. 10.1001/jama.2015.4189 26024502

[B35] Zarbock A. Weiss R. Albert F. Rutledge K. Kellum J. A. Bellomo R. (2023). Epidemiology of surgery associated acute kidney injury (EPIS-AKI): a prospective international observational multi-center clinical study. Intensive Care Med. 49 (12), 1441–1455. 10.1007/s00134-023-07169-7 37505258 PMC10709241

