# Supplementary material for: Development and validation of a risk prediction model for perioperative acute kidney injury in non-cardiac and non-urological surgery patients: a retrospective cohort study
Source: Front Physiol. 2025 Jul 17;16:1628450. doi: 10.3389/fphys.2025.1628450 (PMC12310588; doi:10.3389/fphys.2025.1628450)
Supplement: Supplementary file 1 [file Table1.pdf]

Table 1 Baseline demographic and clinical characteristics of included patients with or without perioperative acute kidney injury.

Variable		Total (n = 40520)	Acute kidney injury		*P*
			No (n = 39,049)	Yes (n = 1,471)	
Sex, n (%)					<0.001
	Male	20,829 (51%)	20,137 (52%)	692 (47%)	
	Female	19,691 (49%)	18,912 (48%)	779 (53%)	
Age, (Median [Q1, Q3]), yr		70.0 (67.0, 74.0)	70.0 (67.0, 74.0)	72.0 (68.0, 78.0)	<0.001
BMI, (Median [Q1, Q3]), kg/m^2		24.4 (22.1, 26.8)	24.4 (22.2, 26.8)	23.7 (21.5, 26.3)	<0.001
ASA classification, n (%)					<0.001
	ASA I	0 (0%)	0 (0%)	0 (0%)	
	ASA II	347 (0.9%)	345 (0.9%)	2 (0.1%)	
	ASA III	27,245 (67%)	26,623 (68%)	622 (42%)	
	ASA IV	12,928 (32%)	12,081 (31%)	847 (58%)	
Duration of surgery, (Median [Q1, Q3]), minute		155 (99, 235)	155 (95, 230)	210 (145, 297)	<0.001
Duration of anesthesia, (Median [Q1, Q3]), minute		175 (113, 255)	170 (110, 250)	230 (160, 320)	<0.001
Anesthesia type, n (%)					<0.001
Simple general anesthesia		25,512 (63%)	24,858 (64%)	654 (44%)	

General anesthesia combined with nerve block		15,008 (37%)	14,191 (36%)	817 (56%)	
Emergency surgery, n (%)					<0.001
	No	35,211 (87%)	34,046 (87%)	1,165 (79%)	
	Yes	5,309 (13%)	5,003 (13%)	306 (21%)	
Cardiovascular disease, n (%)					<0.001
	No	36,962 (91%)	35,723 (91%)	1,239 (84%)	
	Yes	3,558 (8.8%)	3,326 (8.5%)	232 (16%)	
Chronic kidney disease, n (%)					<0.001
	No	38,295 (95%)	37,022 (95%)	1,273 (87%)	
	Yes	2,225 (5.5%)	2,027 (5.2%)	198 (13%)	
Renal surgery, n (%)					<0.001
	No	40,184 (99%)	38,744 (99%)	1,440 (98%)	
	Yes	336 (0.8%)	305 (0.8%)	31 (2.1%)	
Hypertension, n (%)					<0.001
	No	31,998 (79%)	30,934 (79%)	1,064 (72%)	
	Yes	8,522 (21%)	8,115 (21%)	407 (28%)	
Liver cirrhosis, n (%)					<0.001

	No	40,147 (99%)	38,719 (99%)	1,428 (97%)	
	Yes	373 (0.9%)	330 (0.8%)	43 (2.9%)	
Preoperative nephrotoxic antibiotic use, n (%)					0.3
	No	39,065 (96%)	37,639 (96%)	1,426 (97%)	
	Yes	1,455 (3.6%)	1,410 (3.6%)	45 (3.1%)	
Child-Pugh score, (Median [Q1, Q3])		5.00 (4.00, 5.00)	5.00 (4.00, 5.00)	5.00 (5.00, 6.00)	<0.001
Preoperative lipid-lowering drugs, n (%)					<0.001
	No	35,708 (88%)	34,464 (88%)	1,244 (85%)	
	Yes	4,812 (12%)	4,585 (12%)	227 (15%)	
Hepatic encephalopathy, n (%)					>0.9
	No	40,519 (100%)	39,048 (100%)	1,471 (100%)	
	Yes	1 (<0.1%)	1 (<0.1%)	0 (0%)	
Ascites, n (%)					<0.001
	No	39,237 (97%)	37,864 (97%)	1,373 (93%)	
	Yes	1,283 (3.2%)	1,185 (3.0%)	98 (6.7%)	
Diabetes, n (%)					<0.001
	No	35,702 (88%)	34,521 (88%)	1,181 (80%)	

	Yes	4,818 (12%)	4,528 (12%)	290 (20%)	
Renal insufficiency, n (%)					<0.001
	No	40,323 (100%)	38,884 (100%)	1,439 (98%)	
	Yes	197 (0.5%)	165 (0.4%)	32 (2.2%)	
Maximum preoperative urine protein within 90 days, n(%)					
	0	38,120 (94%)	36,852 (94%)	1,268 (86%)	
	1	1,410 (3.5%)	1,317 (3.4%)	93 (6.3%)	
	2	711 (1.8%)	640 (1.6%)	71 (4.8%)	
	3	260 (0.6%)	224 (0.6%)	36 (2.4%)	
	4	9 (<0.1%)	6 (<0.1%)	3 (0.2%)	
	5	2 (<0.1%)	2 (<0.1%)	0 (0%)	
	6	4 (<0.1%)	4 (<0.1%)	0 (0%)	
	7	2 (<0.1%)	2 (<0.1%)	0 (0%)	
	8	1 (<0.1%)	1 (<0.1%)	0 (0%)	
	9	1 (<0.1%)	1 (<0.1%)	0 (0%)	
Last preoperative total bilirubin level, n (%)					<0.001
	0	6,914 (17%)	6,745 (17%)	169 (11%)	

	1	31,612 (78%)	30,542 (78%)	1,070 (73%)	
	2	496 (1.2%)	463 (1.2%)	33 (2.2%)	
	3	1,498 (3.7%)	1,299 (3.3%)	199 (14%)	
Last preoperative albumin level, n (%)					<0.001
	0	7,711 (19%)	7,539 (19%)	172 (12%)	
	1	24,593 (61%)	23,815 (61%)	778 (53%)	
	2	6,958 (17%)	6,535 (17%)	423 (29%)	
	3	1,258 (3.1%)	1,160 (3.0%)	98 (6.7%)	
Last preoperative prothrombin time, n (%)					0.003
	No	12,809 (32%)	12,325 (32%)	484 (33%)	
	Yes	27,223 (67%)	26,268 (67%)	955 (65%)	
	2	291 (0.7%)	271 (0.7%)	20 (1.4%)	
	3	197 (0.5%)	185 (0.5%)	12 (0.8%)	
Preoperative diuretics, n (%)					<0.001
	No	35,301 (87%)	34,250 (88%)	1,051 (71%)	
	Yes	5,219 (13%)	4,799 (12%)	420 (29%)	
Smoking history, n (%)					<0.001

	No	35,302 (87%)	34,084 (87%)	1,218 (83%)	
	Yes	5,218 (13%)	4,965 (13%)	253 (17%)	
Alcohol history, n (%)					<0.001
	No	35,030 (86%)	33,814 (87%)	1,216 (83%)	
	Yes	5,490 (14%)	5,235 (13%)	255 (17%)	
Coronary artery disease, n (%)					<0.001
	No	36,795 (91%)	35,577 (91%)	1,218 (83%)	
	Yes	3,725 (9.2%)	3,472 (8.9%)	253 (17%)	
Angina, n (%)					>0.9
	No	40,397 (100%)	38,930 (100%)	1,467 (100%)	
	Yes	123 (0.3%)	119 (0.3%)	4 (0.3%)	
Valvular heart disease, n (%)					0.005
	No	40,161 (99%)	38,713 (99%)	1,448 (98%)	
	Yes	359 (0.9%)	336 (0.9%)	23 (1.6%)	
Myocardial infarction, n (%)					0.001
	No	40,208 (99%)	38,759 (99%)	1,449 (99%)	
	Yes	312 (0.8%)	290 (0.7%)	22 (1.5%)	

Heart failure, n (%)					<0.001
	No	40,438 (100%)	38,984 (100%)	1,454 (99%)	
	Yes	82 (0.2%)	65 (0.2%)	17 (1.2%)	
Arrhythmia, n (%)					<0.001
	No	39,627 (98%)	38,217 (98%)	1,410 (96%)	
	Yes	893 (2.2%)	832 (2.1%)	61 (4.1%)	
Atrial fibrillation, n (%)					<0.001
	No	40,093 (99%)	38,660 (99%)	1,433 (97%)	
	Yes	427 (1.1%)	389 (1.0%)	38 (2.6%)	
Coronary stent implantation, n (%)					<0.001
	No	39,882 (98%)	38,459 (98%)	1,423 (97%)	
	Yes	638 (1.6%)	590 (1.5%)	48 (3.3%)	
Cardiac surgery, n (%)					<0.001
	No	5,530 (14%)	5,530 (14%)	0 (0%)	
	Yes	34,990 (86%)	33,519 (86%)	1,471 (100%)	
Peripheral vascular disease, n (%)					<0.001
	No	36,851 (91%)	35,549 (91%)	1,302 (89%)	

	Yes	3,669 (9.1%)	3,500 (9.0%)	169 (11%)	
Chronic obstructive pulmonary disease, n (%)					<0.001
	No	39,853 (98%)	38,430 (98%)	1,423 (97%)	
	Yes	667 (1.6%)	619 (1.6%)	48 (3.3%)	
Dialysis, n (%)					0.2
	No	39,127 (97%)	37,715 (97%)	1,412 (96%)	
	Yes	1,393 (3.4%)	1,334 (3.4%)	59 (4.0%)	
History of cerebrovascular disease, n (%)					<0.001
	No	36,349 (90%)	35,135 (90%)	1,214 (83%)	
	Yes	4,171 (10%)	3,914 (10%)	257 (17%)	
Transient ischemic attack, n (%)					0.5
	No	40,460 (100%)	38,992 (100%)	1,468 (100%)	
	Yes	60 (0.1%)	57 (0.1%)	3 (0.2%)	
Stroke, n (%)					<0.001
	No	36,749 (91%)	35,509 (91%)	1,240 (84%)	
	Yes	3,771 (9.3%)	3,540 (9.1%)	231 (16%)	
Paraplegia, n (%)					<0.001

	No	40,379 (100%)	38,921 (100%)	1,458 (99%)	
	Yes	141 (0.3%)	128 (0.3%)	13 (0.9%)	
Malignancy, n (%)					<0.001
	No	33,972 (84%)	32,812 (84%)	1,160 (79%)	
	Yes	6,548 (16%)	6,237 (16%)	311 (21%)	
Preoperative white blood cell count, (Median [Q1, Q3]), 10^9/L		6.55 (5.18, 8.28)	6.56 (5.20, 8.28)	6.26 (4.89, 8.25)	<0.001
Preoperative red blood cell count, (Median [Q1, Q3]), 10^12/L		4.10 (3.72, 4.42)	4.10 (3.73, 4.42)	3.88 (3.39, 4.30)	<0.001
Preoperative neutrophil count, (Median [Q1, Q3]), 10^9/L		4.16 (3.05, 5.70)	4.17 (3.06, 5.69)	4.07 (2.94, 5.98)	>0.9
Preoperative lymphocyte count, (Median [Q1, Q3]), 10^9/L		1.55 (1.18, 1.95)	1.56 (1.20, 1.95)	1.30 (0.89, 1.74)	<0.001
Preoperative monocyte count, (Median [Q1, Q3]), 10^9/L		0.41 (0.30, 0.52)	0.41 (0.30, 0.52)	0.42 (0.30, 0.57)	<0.001
Preoperative eosinophil count, (Median [Q1, Q3]), 10^9/L		0.10 (0.05, 0.16)	0.10 (0.05, 0.16)	0.09 (0.04, 0.17)	0.001
Preoperative basophil count, (Median [Q1, Q3]), 10^9/L		0.020 (0.010, 0.031)	0.020 (0.010, 0.031)	0.020 (0.010, 0.040)	0.038
Preoperative mean corpuscular volume, (Median [Q1, Q3]), fL		92.5 (89.7, 95.3)	92.5 (89.7, 95.3)	92.6 (89.2, 96.2)	0.2
Preoperative mean corpuscular hemoglobin, (Median [Q1, Q3]), pg		30.60 (29.60, 31.60)	30.60 (29.60, 31.60)	30.60 (29.50, 31.90)	0.069
Preoperative mean corpuscular hemoglobin concentration, (Median [Q1, Q3]), g/dL		330 (323, 337)	330 (323, 337)	330 (322, 338)	0.5
Preoperative red cell distribution width, (Median [Q1, Q3]), %		43 (40, 46)	42 (40, 45)	44 (40, 49)	<0.001
Preoperative platelet count, (Median [Q1, Q3]), 10^9/L		213 (174, 253)	213 (175, 253)	201 (151, 254)	<0.001

Preoperative mean platelet volume, (Median [Q1, Q3]), fL	10.19 (9.50, 10.90)	10.17 (9.50, 10.90)	10.50 (9.70, 11.50)	<0.001
Preoperative plateletcrit, (Median [Q1, Q3]), %	28 (24, 34)	28 (24, 33)	31 (25, 38)	<0.001
Preoperative platelet distribution width, (Median [Q1, Q3]), %	12.00 (11.00, 14.00)	12.00 (11.00, 14.00)	12.80 (11.20, 15.00)	<0.001
Preoperative hemoglobin level, (Median [Q1, Q3]), g/L	125 (113, 135)	125 (113, 135)	118 (103, 131)	<0.001
Preoperative hematocrit level, (Median [Q1, Q3]), %	33 (0, 39)	33 (0, 39)	30 (0, 37)	<0.001
Preoperative serum creatinine, (Median [Q1, Q3]), μmol/L	58 (50, 68)	58 (50, 67)	60 (47, 80)	<0.001
Preoperative serum albumin, (Median [Q1, Q3]), g/L	38.1 (35.2, 41.2)	38.2 (35.3, 41.2)	36.2 (32.9, 39.5)	<0.001
Preoperative serum total protein, (Median [Q1, Q3]), g/L	64 (60, 69)	64 (61, 69)	63 (58, 67)	<0.001
Preoperative alanine aminotransferase, (Median [Q1, Q3]), U/L	19 (13, 28)	19 (13, 28)	21 (13, 46)	<0.001
Preoperative aspartate aminotransferase, (Median [Q1, Q3]), U/L	22 (17, 28)	22 (17, 28)	24 (18, 44)	<0.001
Preoperative total bilirubin, (Median [Q1, Q3]), μmol/L	11 (8, 15)	11 (8, 15)	12 (8, 23)	<0.001
Preoperative direct bilirubin, (Median [Q1, Q3]), μmol/L	3.5 (2.7, 4.7)	3.5 (2.6, 4.6)	4.1 (2.8, 9.8)	<0.001
Preoperative glucose, (Median [Q1, Q3]), mmol/L	5.39 (4.88, 6.20)	5.39 (4.88, 6.17)	5.66 (4.82, 7.20)	<0.001
Preoperative serum sodium, (Median [Q1, Q3]), mmol/L	141.0 (139.0, 142.1)	141.0 (139.0, 142.2)	140.0 (137.0, 142.0)	<0.001
Preoperative serum potassium, (Median [Q1, Q3]), mmol/L	4.11 (3.88, 4.38)	4.11 (3.88, 4.38)	4.03 (3.72, 4.36)	<0.001
Preoperative serum chloride, (Median [Q1, Q3]), mmol/L	104.5 (102.2, 106.4)	104.6 (102.3, 106.4)	103.8 (101.0, 106.6)	<0.001
Preoperative serum calcium, (Median [Q1, Q3]), mmol/L	2.21 (2.11, 2.30)	2.21 (2.12, 2.30)	2.18 (2.07, 2.28)	<0.001

Preoperative thrombin time, (Median [Q1, Q3]), s		16.70 (15.90, 17.70)	16.70 (15.90, 17.70)	16.90 (15.97, 17.90)	<0.001
Preoperative activated partial thromboplastin time (APTT), (Median [Q1, Q3]), s		32.8 (29.4, 36.5)	32.8 (29.5, 36.5)	32.8 (28.3, 36.9)	0.5
Preoperative prothrombin time, (Median [Q1, Q3]), s		12.30 (11.50, 13.10)	12.30 (11.50, 13.10)	12.40 (11.50, 13.30)	<0.001
Preoperative plasma fibrinogen level, (Median [Q1, Q3]), g/L		3.42 (2.84, 4.07)	3.41 (2.84, 4.06)	3.61 (2.91, 4.57)	<0.001
Preoperative international normalized ratio, (Median [Q1, Q3])		0.95 (0.88, 1.04)	0.95 (0.88, 1.04)	0.97 (0.90, 1.07)	<0.001
Preoperative antihypertensive medications, n (%)					<0.001
	No	23,152 (57%)	22,599 (58%)	553 (38%)	
	Yes	17,368 (43%)	16,450 (42%)	918 (62%)	
Preoperative ACE inhibitors, n (%)					<0.001
	No	36,795 (91%)	35,503 (91%)	1,292 (88%)	
	Yes	3,725 (9.2%)	3,546 (9.1%)	179 (12%)	
Preoperative ARB inhibitors, n (%)					<0.001
	No	37,400 (92%)	36,098 (92%)	1,302 (89%)	
	Yes	3,120 (7.7%)	2,951 (7.6%)	169 (11%)	
Preoperative calcium channel blockers, n (%)					<0.001
	No	30,319 (75%)	29,382 (75%)	937 (64%)	
	Yes	10,201 (25%)	9,667 (25%)	534 (36%)	

Preoperative metoprolol, n (%)					<0.001
	No	38,304 (95%)	36,972 (95%)	1,332 (91%)	
	Yes	2,216 (5.5%)	2,077 (5.3%)	139 (9.4%)	
Preoperative steroids, n (%)					0.5
	No	32,020 (79%)	30,869 (79%)	1,151 (78%)	
	Yes	8,500 (21%)	8,180 (21%)	320 (22%)	
Preoperative statins or fibrates, n (%)					<0.001
	No	35,708 (88%)	34,464 (88%)	1,244 (85%)	
	Yes	4,812 (12%)	4,585 (12%)	227 (15%)	
Preoperative anticoagulants, n (%)					<0.001
	No	33,566 (83%)	32,501 (83%)	1,065 (72%)	
	Yes	6,954 (17%)	6,548 (17%)	406 (28%)	
Preoperative antiplatelet medications, n (%)					<0.001
	No	35,526 (88%)	34,295 (88%)	1,231 (84%)	
	Yes	4,994 (12%)	4,754 (12%)	240 (16%)	
Preoperative β-blockers, n (%)					<0.001
	No	36,979 (91%)	35,715 (91%)	1,264 (86%)	

	Yes	3,541 (8.7%)	3,334 (8.5%)	207 (14%)	
Preoperative calcium ion channel blockers, n (%)					<0.001
	No	30,319 (75%)	29,382 (75%)	937 (64%)	
	Yes	10,201 (25%)	9,667 (25%)	534 (36%)	
Perioperative non-steroidal anti-inflammatory drugs, n (%)					<0.001
	No	6,712 (17%)	6,660 (17%)	52 (3.5%)	
	Yes	33,808 (83%)	32,389 (83%)	1,419 (96%)	
Preoperative hypoglycemic medications, n (%)					<0.001
	No	36,124 (89%)	34,891 (89%)	1,233 (84%)	
	Yes	4,396 (11%)	4,158 (11%)	238 (16%)	
Preoperative insulin, n (%)					<0.001
	No	32,876 (81%)	31,897 (82%)	979 (67%)	
	Yes	7,644 (19%)	7,152 (18%)	492 (33%)	
Preoperative systolic blood pressure, (Median [Q1, Q3]), mmHg		132 (124, 141)	132 (124, 141)	134 (122, 146)	0.002
Preoperative diastolic blood pressure, (Median [Q1, Q3]), mmHg		78 (73, 84)	78 (73, 84)	77 (71, 85)	<0.001
Aspirin use during this hospitalization, n (%)					<0.001
	No	34,354 (85%)	33,182 (85%)	1,172 (80%)	

	Yes	6,166 (15%)	5,867 (15%)	299 (20%)	
Preoperative aspirin use, n (%)					0.004
	No	40,302 (99%)	38,831 (99%)	1,471 (100%)	
	Yes	218 (0.5%)	218 (0.6%)	0 (0%)	
Clopidogrel use during this hospitalization, n (%)					<0.001
	No	39,221 (97%)	37,838 (97%)	1,383 (94%)	
	Yes	1,299 (3.2%)	1,211 (3.1%)	88 (6.0%)	
Preoperative clopidogrel use, n (%)					0.011
	No	40,383 (100%)	38,912 (100%)	1,471 (100%)	
	Yes	137 (0.3%)	137 (0.4%)	0 (0%)	
Heparin use during this hospitalization, n (%)					<0.001
	No	11,048 (27%)	10,993 (28%)	55 (3.7%)	
	Yes	29,472 (73%)	28,056 (72%)	1,416 (96%)	
Preoperative heparin use, n (%)					<0.001
	No	40,039 (99%)	38,568 (99%)	1,471 (100%)	
	Yes	481 (1.2%)	481 (1.2%)	0 (0%)	
Preoperative dextran use, n (%)					0.08

	No	40,475 (100%)	39,008 (100%)	1,467 (100%)
	Yes	45 (0.1%)	41 (0.1%)	4 (0.3%)

Abbreviations: ASA, American Society of Anesthesiologists; ARB, Angiotensin II Receptor Blockers; ACEI, Angiotensin-Converting Enzyme Inhibitors.
