# Supplementary material for: Development and validation of a risk prediction model for perioperative acute kidney injury in non-cardiac and non-urological surgery patients: a retrospective cohort study
Source: Front Physiol. 2025 Jul 17;16:1628450. doi: 10.3389/fphys.2025.1628450 (PMC12310588; doi:10.3389/fphys.2025.1628450)
Supplement: Supplementary file 2 [file Table2.pdf]

Table 2 Baseline demographic and clinical characteristics of included patients between training cohort and validation cohort

Variable		Total (n = 40520)	Training cohort (n = 28,364)	Validation cohort (n = 12,156)	*P*
AKI, n (%)					>0.9
	No	39,049 (96%)	27,335 (96%)	11,714 (96%)	
	Yes	1,471 (3.6%)	1,029 (3.6%)	442 (3.6%)	
Sex, n (%)					0.2
	Male	20,829 (51%)	14,514 (51%)	6,315 (52%)	
	Female	19,691 (49%)	13,850 (49%)	5,841 (48%)	
Age, (Median [Q1, Q3]), yr		70.0 (67.0, 74.0)	70.0 (67.0, 74.0)	70.0 (67.0, 74.0)	>0.9
BMI, (Median [Q1, Q3]), kg/m^2		24.4 (22.1, 26.8)	24.4 (22.2, 26.8)	24.3 (22.0, 26.7)	0.006
ASA classification, n (%)					0.6
	ASA I	0 (0%)	0 (0%)	0 (0%)	
	ASA II	347 (0.9%)	248 (0.9%)	99 (0.8%)	
	ASA III	27,245 (67%)	19,035 (67%)	8,210 (68%)	
	ASA IV	12,928 (32%)	9,081 (32%)	3,847 (32%)	
Duration of surgery, (Median [Q1, Q3]), minute		155 (99, 235)	155 (100, 235)	155 (97, 235)	0.8
Duration of anesthesia, (Median [Q1, Q3]), minute		175 (113, 255)	175 (113, 255)	175 (113, 255)	0.8

Anesthesia type, n (%)					0.032
Simple general anesthesia		25,512 (63%)	17,954 (63%)	7,558 (62%)	
General anesthesia combined with nerve block		15,008 (37%)	10,410 (37%)	4,598 (38%)	
Emergency surgery, n (%)					0.5
	No	35,211 (87%)	24,627 (87%)	10,584 (87%)	
	Yes	5,309 (13%)	3,737 (13%)	1,572 (13%)	
Cardiovascular disease, n (%)					0.8
	No	36,962 (91%)	25,881 (91%)	11,081 (91%)	
	Yes	3,558 (8.8%)	2,483 (8.8%)	1,075 (8.8%)	
Chronic kidney disease, n (%)					>0.9
	No	38,295 (95%)	26,807 (95%)	11,488 (95%)	
	Yes	2,225 (5.5%)	1,557 (5.5%)	668 (5.5%)	
Renal surgery, n (%)					0.04
	No	40,184 (99%)	28,146 (99%)	12,038 (99%)	
	Yes	336 (0.8%)	218 (0.8%)	118 (1.0%)	
Hypertension, n (%)					0.5
	No	31,998 (79%)	22,375 (79%)	9,623 (79%)	

	Yes	8,522 (21%)	5,989 (21%)	2,533 (21%)	
Liver cirrhosis, n (%)					0.2
	No	40,147 (99%)	28,115 (99%)	12,032 (99%)	
	Yes	373 (0.9%)	249 (0.9%)	124 (1.0%)	
Preoperative nephrotoxic antibiotic use, n (%)					0.4
	No	39,065 (96%)	27,359 (96%)	11,706 (96%)	
	Yes	1,455 (3.6%)	1,005 (3.5%)	450 (3.7%)	
Child-Pugh score, (Median [Q1, Q3])		5.00 (4.00, 5.00)	5.00 (4.00, 5.00)	5.00 (4.00, 5.00)	0.083
Preoperative lipid-lowering drugs, n (%)					0.4
	No	35,708 (88%)	24,968 (88%)	10,740 (88%)	
	Yes	4,812 (12%)	3,396 (12%)	1,416 (12%)	
Hepatic encephalopathy, n (%)					>0.9
	No	40,519 (100%)	28,363 (100%)	12,156 (100%)	
	Yes	1 (<0.1%)	1 (<0.1%)	0 (0%)	
Ascites, n (%)					0.022
	No	39,237 (97%)	27,503 (97%)	11,734 (97%)	
	Yes	1,283 (3.2%)	861 (3.0%)	422 (3.5%)	

Diabetes, n (%)					0.8
	No	35,702 (88%)	24,984 (88%)	10,718 (88%)	
	Yes	4,818 (12%)	3,380 (12%)	1,438 (12%)	
Renal insufficiency, n (%)					0.9
	No	40,323 (100%)	28,227 (100%)	12,096 (100%)	
	Yes	197 (0.5%)	137 (0.5%)	60 (0.5%)	
Maximum preoperative urine protein within 90 days, n(%)					
	0	38,120 (94%)	26,687 (94%)	11,433 (94%)	
	1	1,410 (3.5%)	1,000 (3.5%)	410 (3.4%)	
	2	711 (1.8%)	476 (1.7%)	235 (1.9%)	
	3	260 (0.6%)	186 (0.7%)	74 (0.6%)	
	4	9 (<0.1%)	7 (<0.1%)	2 (<0.1%)	
	5	2 (<0.1%)	1 (<0.1%)	1 (<0.1%)	
	6	4 (<0.1%)	3 (<0.1%)	1 (<0.1%)	
	7	2 (<0.1%)	2 (<0.1%)	0 (0%)	
	8	1 (<0.1%)	1 (<0.1%)	0 (0%)	
	9	1 (<0.1%)	1 (<0.1%)	0 (0%)	

Last preoperative total bilirubin level, n (%)					0.5
	0	6,914 (17%)	4,863 (17%)	2,051 (17%)	
	1	31,612 (78%)	22,114 (78%)	9,498 (78%)	
	2	496 (1.2%)	357 (1.3%)	139 (1.1%)	
	3	1,498 (3.7%)	1,030 (3.6%)	468 (3.8%)	
Last preoperative albumin level, n (%)					0.5
	0	7,711 (19%)	5,439 (19%)	2,272 (19%)	
	1	24,593 (61%)	17,216 (61%)	7,377 (61%)	
	2	6,958 (17%)	4,829 (17%)	2,129 (18%)	
	3	1,258 (3.1%)	880 (3.1%)	378 (3.1%)	
Last preoperative prothrombin time, n (%)					0.5
	No	12,809 (32%)	8,998 (32%)	3,811 (31%)	
	Yes	27,223 (67%)	19,032 (67%)	8,191 (67%)	
	2	291 (0.7%)	194 (0.7%)	97 (0.8%)	
	3	197 (0.5%)	140 (0.5%)	57 (0.5%)	
Preoperative diuretics, n (%)					>0.9
	No	35,301 (87%)	24,709 (87%)	10,592 (87%)	

	Yes	5,219 (13%)	3,655 (13%)	1,564 (13%)	
Smoking history, n (%)					0.4
	No	35,302 (87%)	24,685 (87%)	10,617 (87%)	
	Yes	5,218 (13%)	3,679 (13%)	1,539 (13%)	
Alcohol history, n (%)					0.5
	No	35,030 (86%)	24,500 (86%)	10,530 (87%)	
	Yes	5,490 (14%)	3,864 (14%)	1,626 (13%)	
Coronary artery disease, n (%)					>0.9
	No	36,795 (91%)	25,759 (91%)	11,036 (91%)	
	Yes	3,725 (9.2%)	2,605 (9.2%)	1,120 (9.2%)	
Angina, n (%)					0.7
	No	40,397 (100%)	28,276 (100%)	12,121 (100%)	
	Yes	123 (0.3%)	88 (0.3%)	35 (0.3%)	
Valvular heart disease, n (%)					0.9
	No	40,161 (99%)	28,114 (99%)	12,047 (99%)	
	Yes	359 (0.9%)	250 (0.9%)	109 (0.9%)	
Myocardial infarction, n (%)					0.07

	No	40,208 (99%)	28,131 (99%)	12,077 (99%)	
	Yes	312 (0.8%)	233 (0.8%)	79 (0.6%)	
Heart failure, n (%)					>0.9
	No	40,438 (100%)	28,307 (100%)	12,131 (100%)	
	Yes	82 (0.2%)	57 (0.2%)	25 (0.2%)	
Arrhythmia, n (%)					0.7
	No	39,627 (98%)	27,734 (98%)	11,893 (98%)	
	Yes	893 (2.2%)	630 (2.2%)	263 (2.2%)	
Atrial fibrillation, n (%)					0.7
	No	40,093 (99%)	28,069 (99%)	12,024 (99%)	
	Yes	427 (1.1%)	295 (1.0%)	132 (1.1%)	
Coronary stent implantation, n (%)					0.9
	No	39,882 (98%)	27,919 (98%)	11,963 (98%)	
	Yes	638 (1.6%)	445 (1.6%)	193 (1.6%)	
Cardiac surgery, n (%)					0.072
	No	5,530 (14%)	3,928 (14%)	1,602 (13%)	
	Yes	34,990 (86%)	24,436 (86%)	10,554 (87%)	

Peripheral vascular disease, n (%)					0.3
	No	36,851 (91%)	25,769 (91%)	11,082 (91%)	
	Yes	3,669 (9.1%)	2,595 (9.1%)	1,074 (8.8%)	
Chronic obstructive pulmonary disease, n (%)					0.4
	No	39,853 (98%)	27,888 (98%)	11,965 (98%)	
	Yes	667 (1.6%)	476 (1.7%)	191 (1.6%)	
Dialysis, n (%)					0.8
	No	39,127 (97%)	27,385 (97%)	11,742 (97%)	
	Yes	1,393 (3.4%)	979 (3.5%)	414 (3.4%)	
History of cerebrovascular disease, n (%)					0.4
	No	36,349 (90%)	25,419 (90%)	10,930 (90%)	
	Yes	4,171 (10%)	2,945 (10%)	1,226 (10%)	
Transient ischemic attack, n (%)					0.4
	No	40,460 (100%)	28,325 (100%)	12,135 (100%)	
	Yes	60 (0.1%)	39 (0.1%)	21 (0.2%)	
Stroke, n (%)					0.4
	No	36,749 (91%)	25,704 (91%)	11,045 (91%)	

	Yes	3,771 (9.3%)	2,660 (9.4%)	1,111 (9.1%)	
Paraplegia, n (%)					0.6
	No	40,379 (100%)	28,268 (100%)	12,111 (100%)	
	Yes	141 (0.3%)	96 (0.3%)	45 (0.4%)	
Malignancy, n (%)					0.4
	No	33,972 (84%)	23,753 (84%)	10,219 (84%)	
	Yes	6,548 (16%)	4,611 (16%)	1,937 (16%)	
Preoperative white blood cell count, (Median [Q1, Q3]), 10^9/L		6.55 (5.18, 8.28)	6.57 (5.19, 8.30)	6.50 (5.15, 8.23)	0.09
Preoperative red blood cell count, (Median [Q1, Q3]), 10^12/L		4.10 (3.72, 4.42)	4.10 (3.72, 4.42)	4.10 (3.71, 4.40)	0.12
Preoperative neutrophil count, (Median [Q1, Q3]), 10^9/L		4.16 (3.05, 5.70)	4.17 (3.06, 5.71)	4.14 (3.03, 5.66)	0.2
Preoperative lymphocyte count, (Median [Q1, Q3]), 10^9/L		1.55 (1.18, 1.95)	1.55 (1.18, 1.95)	1.55 (1.19, 1.94)	0.4
Preoperative monocyte count, (Median [Q1, Q3]), 10^9/L		0.41 (0.30, 0.52)	0.41 (0.30, 0.52)	0.41 (0.30, 0.51)	0.3
Preoperative eosinophil count, (Median [Q1, Q3]), 10^9/L		0.10 (0.05, 0.16)	0.10 (0.05, 0.16)	0.10 (0.05, 0.16)	0.12
Preoperative basophil count, (Median [Q1, Q3]), 10^9/L		0.020 (0.010, 0.031)	0.020 (0.010, 0.031)	0.020 (0.010, 0.036)	0.15
Preoperative mean corpuscular volume, (Median [Q1, Q3]), fL		92.5 (89.7, 95.3)	92.5 (89.7, 95.3)	92.6 (89.8, 95.4)	0.089
Preoperative mean corpuscular hemoglobin, (Median [Q1, Q3]), pg		30.60 (29.60, 31.60)	30.60 (29.50, 31.60)	30.60 (29.60, 31.60)	0.14
Preoperative mean corpuscular hemoglobin concentration (MCHC),(Median [Q1, Q3]), g/dL		330 (323, 337)	330 (323, 337)	330 (323, 337)	0.6

Preoperative red cell distribution width, (Median [Q1, Q3]), %	43 (40, 46)	42 (40, 45)	43 (40, 46)	0.077
Preoperative platelet count, (Median [Q1, Q3]), 10^9/L	213 (174, 253)	213 (174, 253)	214 (175, 253)	0.3
Preoperative mean platelet volume, (Median [Q1, Q3]), fL	10.19 (9.50, 10.90)	10.19 (9.50, 10.90)	10.20 (9.50, 11.00)	0.2
Preoperative plateletcrit, (Median [Q1, Q3]), %	28 (24, 34)	28 (24, 34)	28 (24, 34)	0.5
Preoperative platelet distribution width, (Median [Q1, Q3]), %	12.00 (11.00, 14.00)	12.00 (11.00, 14.00)	12.00 (11.00, 14.00)	0.6
Preoperative hemoglobin level, (Median [Q1, Q3]), g/L	125 (113, 135)	125 (113, 135)	125 (113, 135)	0.5
Preoperative hematocrit level, (Median [Q1, Q3]), %	33 (0, 39)	33 (0, 39)	33 (0, 39)	0.017
Preoperative serum creatinine, (Median [Q1, Q3]), μmol/L	58 (50, 68)	58 (50, 68)	58 (50, 67)	0.069
Preoperative serum albumin, (Median [Q1, Q3]), g/L	38.1 (35.2, 41.2)	38.1 (35.3, 41.2)	38.1 (35.2, 41.2)	0.8
Preoperative serum total protein, (Median [Q1, Q3]), g/L	64 (60, 69)	64 (60, 69)	64 (61, 69)	0.7
Preoperative alanine aminotransferase, (Median [Q1, Q3]), U/L	19 (13, 28)	19 (13, 28)	19 (13, 28)	0.14
Preoperative aspartate aminotransferase, (Median [Q1, Q3]), U/L	22 (17, 28)	22 (17, 28)	22 (17, 28)	0.6
Preoperative total bilirubin, (Median [Q1, Q3]), μmol/L	11 (8, 15)	11 (8, 15)	11 (8, 15)	0.4
Preoperative direct bilirubin, (Median [Q1, Q3]), μmol/L	3.5 (2.7, 4.7)	3.5 (2.7, 4.7)	3.5 (2.6, 4.7)	>0.9
Preoperative glucose, (Median [Q1, Q3]), mmol/L	5.39 (4.88, 6.20)	5.40 (4.89, 6.20)	5.38 (4.86, 6.20)	0.07
Preoperative serum sodium, (Median [Q1, Q3]), mmol/L	141.0 (139.0, 142.1)	141.0 (139.0, 142.1)	141.0 (139.0, 142.2)	0.5
Preoperative serum potassium, (Median [Q1, Q3]), mmol/L	4.11 (3.88, 4.38)	4.11 (3.88, 4.38)	4.11 (3.88, 4.39)	0.3

Preoperative serum chloride, (Median [Q1, Q3]), mmol/L		104.5 (102.2, 106.4)	104.5 (102.3, 106.4)	104.6 (102.1, 106.5)	0.5
Preoperative serum calcium, (Median [Q1, Q3]), mmol/L		2.21 (2.11, 2.30)	2.21 (2.12, 2.30)	2.20 (2.11, 2.30)	0.2
Preoperative thrombin time, (Median [Q1, Q3]), s		16.70 (15.90, 17.70)	16.70 (15.90, 17.70)	16.70 (15.90, 17.70)	0.6
Preoperative activated partial thromboplastin time (APTT), (Median [Q1, Q3]), s		32.8 (29.4, 36.5)	32.8 (29.4, 36.4)	32.8 (29.4, 36.5)	0.8
Preoperative prothrombin time, (Median [Q1, Q3]), s		12.30 (11.50, 13.10)	12.30 (11.50, 13.10)	12.30 (11.50, 13.10)	0.6
Preoperative plasma fibrinogen level, (Median [Q1, Q3]), g/L		3.42 (2.84, 4.07)	3.41 (2.84, 4.07)	3.43 (2.84, 4.07)	0.4
Preoperative international normalized ratio, (Median [Q1, Q3])		0.95 (0.88, 1.04)	0.95 (0.88, 1.04)	0.95 (0.88, 1.04)	>0.9
Preoperative antihypertensive medications, n (%)					0.7
	No	23,152 (57%)	16,187 (57%)	6,965 (57%)	
	Yes	17,368 (43%)	12,177 (43%)	5,191 (43%)	
Preoperative ACE inhibitors, n (%)					0.3
	No	36,795 (91%)	25,729 (91%)	11,066 (91%)	
	Yes	3,725 (9.2%)	2,635 (9.3%)	1,090 (9.0%)	
Preoperative ARB inhibitors, n (%)					0.3
	No	37,400 (92%)	26,153 (92%)	11,247 (93%)	
	Yes	3,120 (7.7%)	2,211 (7.8%)	909 (7.5%)	
Preoperative calcium channel blockers, n (%)					0.4

	No	30,319 (75%)	21,193 (75%)	9,126 (75%)	
	Yes	10,201 (25%)	7,171 (25%)	3,030 (25%)	
Preoperative metoprolol, n (%)					0.088
	No	38,304 (95%)	26,777 (94%)	11,527 (95%)	
	Yes	2,216 (5.5%)	1,587 (5.6%)	629 (5.2%)	
Preoperative steroids, n (%)					0.14
	No	32,020 (79%)	22,358 (79%)	9,662 (79%)	
	Yes	8,500 (21%)	6,006 (21%)	2,494 (21%)	
Preoperative statins or fibrates, n (%)					0.4
	No	35,708 (88%)	24,968 (88%)	10,740 (88%)	
	Yes	4,812 (12%)	3,396 (12%)	1,416 (12%)	
Preoperative anticoagulants, n (%)					0.6
	No	33,566 (83%)	23,480 (83%)	10,086 (83%)	
	Yes	6,954 (17%)	4,884 (17%)	2,070 (17%)	
Preoperative antiplatelet medications, n (%)					0.3
	No	35,526 (88%)	24,839 (88%)	10,687 (88%)	
	Yes	4,994 (12%)	3,525 (12%)	1,469 (12%)	

Preoperative β-blockers, n (%)					0.3
	No	36,979 (91%)	25,857 (91%)	11,122 (91%)	
	Yes	3,541 (8.7%)	2,507 (8.8%)	1,034 (8.5%)	
Preoperative calcium ion channel blockers, n (%)					0.4
	No	30,319 (75%)	21,193 (75%)	9,126 (75%)	
	Yes	10,201 (25%)	7,171 (25%)	3,030 (25%)	
Perioperative non-steroidal anti-inflammatory drugs, n (%)					0.8
	No	6,712 (17%)	4,709 (17%)	2,003 (16%)	
	Yes	33,808 (83%)	23,655 (83%)	10,153 (84%)	
Preoperative hypoglycemic medications, n (%)					0.8
	No	36,124 (89%)	25,280 (89%)	10,844 (89%)	
	Yes	4,396 (11%)	3,084 (11%)	1,312 (11%)	
Preoperative insulin, n (%)					0.4
	No	32,876 (81%)	23,044 (81%)	9,832 (81%)	
	Yes	7,644 (19%)	5,320 (19%)	2,324 (19%)	
Preoperative systolic blood pressure, (Median [Q1, Q3]), mmHg		132 (124, 141)	132 (124, 141)	132 (124, 141)	0.8
Preoperative diastolic blood pressure, (Median [Q1, Q3]), mmHg		78 (73, 84)	78 (73, 84)	78 (73, 84)	0.7

Aspirin use during this hospitalization, n (%)					0.6
	No	34,354 (85%)	24,028 (85%)	10,326 (85%)	
	Yes	6,166 (15%)	4,336 (15%)	1,830 (15%)	
Preoperative aspirin use, n (%)					0.8
	No	40,302 (99%)	28,210 (99%)	12,092 (99%)	
	Yes	218 (0.5%)	154 (0.5%)	64 (0.5%)	
Clopidogrel use during this hospitalization, n (%)					0.7
	No	39,221 (97%)	27,449 (97%)	11,772 (97%)	
	Yes	1,299 (3.2%)	915 (3.2%)	384 (3.2%)	
Preoperative clopidogrel use, n (%)					>0.9
	No	40,383 (100%)	28,268 (100%)	12,115 (100%)	
	Yes	137 (0.3%)	96 (0.3%)	41 (0.3%)	
Heparin use during this hospitalization, n (%)					0.9
	No	11,048 (27%)	7,726 (27%)	3,322 (27%)	
	Yes	29,472 (73%)	20,638 (73%)	8,834 (73%)	
Preoperative heparin use, n (%)					0.6
	No	40,039 (99%)	28,022 (99%)	12,017 (99%)	

	Yes	481 (1.2%)	342 (1.2%)	139 (1.1%)	
Preoperative dextran use, n (%)					0.14
	No	40,475 (100%)	28,328 (100%)	12,147 (100%)	
	Yes	45 (0.1%)	36 (0.1%)	9 (<0.1%)	

Abbreviations: ASA, American Society of Anesthesiologists; ARB, Angiotensin II Receptor Blockers; ACEI, Angiotensin-Converting Enzyme Inhibitors.
